# Supplementary material for: Diagnostic Performance of ACR and Kwak TI-RADS for Benign and Malignant Thyroid Nodules: An Update Systematic Review and Meta-Analysis
Source: Cancers (Basel). 2022 Dec 2;14(23):5961. doi: 10.3390/cancers14235961 (PMC9740871; doi:10.3390/cancers14235961)
Supplement: Supplementary file 1 [file cancers-14-05961-s001.zip › cancers-2018629-supplementary.pdf]

Table S1. Study characteristics.

Study	Design	Total Number of Patients (n)	Age of Patients with Robotic Approach (years, Mean ± SD or Median (Interquartile Range))	Sex (F/M)	Nation	Nodule Size (mm, Mean ± SD or Median (Range))	Tumor Size (cm, Mean ± SD)	Number of Specimen (n)	The Thyroid Imaging Reporting and Data System (TI-RADS)	Reference Test
Yoon 2015 [50]	Retrospective	1241	50.8 ± 13.5	NA	Korea	18.8 ± 10.7	>1.0	1293	Kwak	Cytologically confirm
Ko 2016 [48]	Retrospective	1102	50 (15-85)	254/874	Korea	12.4 ± 7.7	≥0.5	1128	Kwak	Cytologically confirm
Park 2016 [49]	Retrospective	103	51 (22-68)	86/18	Korea	8.9 ± 5.9	All	104	Kwak	Cytologically or pathologically confirm
Yoon 2016 [15]	Retrospective	87	59 ± 12	48/39	Korea	17 ± 13	≥0.5	87	Kwak	Cytologically or pathologically confirm
Grani 2017 [51]	Retrospective	47	54 ± 12	34/13	Italy	NA	All	49	Kwak	Surgical pathology
Chng 2018 [16]	Retrospective	150	54.4 ± 12.4	120/30	Singapore	31 ± 15	>1.0	167	Kwak	Surgical pathology
Ha 2018 [11]	Retrospective	750	49.2 (9-81)	594/156	Korea	15 ±1.1	>0.5	902	ACR	Cytopathologic results of the Bethesda system, and surgical findings
Ahmadi 2019 [17]	Retrospective	186	57 (44-68)	154/32	USA	24 (15-35)	All	202	ACR	Surgical pathology
Gao 2019 [19]	Retrospective	1758	48.5 ± 12.0	1788/756	China	11 ± 7	All	2544	Kwak, ACR	Surgical pathology
Li 2019 [20]	Retrospective	93	55.21 ± 2.95	73/20	China	17.2 ± 1.8	All	93	Kwak	Surgical pathology
Phuttharak 2019 [21]	Retrospective	139	51.6 ± 13.08	87/7	Thailand	21.2 ± 12	All	108	ACR	Cytologically confirm
Shen 2019 [23]	Retrospective	1,568	48 ± 13	1192/420	China	13.59 ± 11.01	>0.5	1,612	Kwak	Cytopathologic results of the Bethesda system, and surgical findings
Shen 2019 [23]	Retrospective	1,568	48 ± 13	1192/420	China	13.59 ± 11.01	>0.5	1,612	ACR	Cytopathologic results of the Bethesda system, and surgical findings
Wu 2019 [73]	Retrospective	894	NA	NA	China	NA	All	1000	ACR	Cytopathologic results of the Bethesda system, and surgical findings
Wu 2019 [74]	Prospective	29	47.6 ± 15.5	NA	China	25.8 ± 13.0	All	43	ACR	Surgical pathology
Zhao 2019 [52]	Retrospective	143	44.56 ± 9.67	111/32	China	7.23 ± 1.52	All	150	Kwak	Surgical pathology
Huang 2020 [24]	Retrospective	260	58 (46-69)	212/48	USA	22 (1.6-3.3)	All	361	ACR	Cytologically confirm
Koc 2020 [25]	Retrospective	460	52 (18-84)	375/85	Turkey	NA	≥0.5	492	ACR	Pathologically confirm
Peng 2020 [26]	Retrospective	230	44.8 ± 11.5	171/59	China	15.6 ± 10.7	All	230	ACR	Cytologically or pathologically confirm
Watkins 2020 [27]	Retrospective	212	58.5 ± 29	161/61	UK	19 ± 19.1	All	218	ACR	Pathologically confirm
Xiao 2020 [28]	Retrospective	792	48.25 ± 12.37	632/160	China	9 (5-19)	All	1940	Kwak, ACR	Cytologically or pathologically confirm
Yang 2020 [29]	Retrospective	92	51.1 ± 13.5	70/22	USA	21 (8-77)	>0.5	92	ACR	Surgical pathology
Yoon 2020 [30]	Retrospective	8364	50.1 ± 13.5	6598/1766	Korea	22.0 ± 12.1	All	8657	Kwak, ACR	Cytologically or pathologically confirm

Zhang 2020 [75]	Retrospective	1271	51.7 ± 12.5	986/285	China	16.4 ± 11.6	All	1271	Kwak, ACR	Cytologically or pathologically confirm
Chen 2021 [47]	Retrospective	125	56.7 ± 15	99/26	Australia	23 (1-130)	All	146	ACR	Cytologically or pathologically confirm
da Silva 2021 [33]	Retrospective	314	51.56 ± 15.4	NA	Brazil	16.9 ± 13.2	All	473	ACR	Cytologically or pathologically confirm
Ha 2021 [34]	Retrospective	5,081	53.2 (19-93)	4,176/905	Korea	20.7 ± 10.8	≥1.0 cm	5,708	ACR	Cytologically or pathologically confirm
Han 2021 [35]	Retrospective	372	49.5 (8-81)	289/83	Korea	17.8 (10.0-73.0)	≥1.0 cm	454	ACR	Cytologically or pathologically confirm
Hekimsoy 2021 [36]	Retrospective	165	49.64 ± 13.50	131/34	Turkey	15.0 (10.0-29.25)	All	251	ACR	Cytologically or pathologically confirm
Huh 2021 [37]	Retrospective	1,301	50.2 ± 13.6	1,062/239	Korea	23.2 ± 12.6	All	1,384	Kwak, ACR	Cytologically or pathologically confirm
Kang 2021 [38]	Retrospective	160	51.2 ± 14.5	145/15	Korea	17 ± 11	All	200	ACR	Cytologically or pathologically confirm
Kim 2021 [53]	Prospective	757	51 (39-61)	587/170	Korea	20 (14-30)	All	760	ACR	Cytologically or pathologically confirm
Liu 2021 [54]	Retrospective	970	46.0 (36-54)	729/241	China	10 (7-15)	All	970	ACR	Pathologically confirm (core needle biopsy)
Na 2021 [39]	Retrospective	NA	NA	NA	Korea	17 (10-100)	≥1.0 cm	NA	ACR	Cytologically or pathologically confirm
Qi 2021 [40]	Retrospective	884	41.83 (10-73)	681/203	China	19.13 (5-64)	All	1096	Kwak, ACR	Cytologically or pathologically confirm
Scappaticcio 2021 [41]	Retrospective	36	15 (11-17)	26/10	Switzerland	13 (10-16)	All	41	ACR	Cytologically or pathologically confirm
Seifert 2021 [42]	Retrospective	849	51 ± 14	604/249	Germany	26 ± 13	All	1211	Kwak, ACR	Cytologically or pathologically confirm
Seminati 2021 [43]	Prospective	448	NA	NA	Italy	NA	All	493	ACR	Cytologically or pathologically confirm
Sharma 2021 [55]	Retrospective	200	(23-74)	164/36	USA	NA	All	200	Kwak, ACR	Cytopathologic results
Shi 2021 [44]	Retrospective	839	54 (14-88)	594/245	China	22 (14-32)	All	846	Kwak, ACR	Cytologically or pathologically confirm
Zhang 2021 [45]	Retrospective	566	47.4 ± 13.4	442/124	China	11.4 ± 5.8	≥5 mm	566	Kwak, ACR	Cytologically or pathologically confirm
Zhu 2021 [46]	Retrospective	1,697	53.2 ± 12.7	1336/361	China	13.1 ± 10.6	All	2,309	ACR	Cytologically or pathologically confirm
Chen 2022 [32]	Retrospective	1978	46.41 ± 12.65	1531/351	China	11.71 ± 8.47	All	1982	ACR	Cytologically or pathologically confirm
Qi 2022 [6]	Retrospective	820	44.5 ± 13.4	619/201	China	13 (11-17)	All	820	Kwak, ACR	Cytologically or pathologically confirm
Thedinger 2022 [76]	Prospective	720	58.7 ± 0.6	538/182	USA	NA	All	949	ACR	Cytologically confirm
Zhang 2022 [77]	Retrospective	509	48.22 ± 12.31	355/154	China	18 (8-34)	All	509	Kwak, ACR	Cytologically or pathologically confirm

SD; standard deviation, ACR; American College of Radiology, NA; not applicable.

Table S2. Methodological quality of the included studies.

Reference	Risk of Bias				Concerns about Application		
	Patient Selection	Index Test	Reference Standard	Flow and Timing	Patient Selection	Index Test	Reference Standard
Yoon 2015 [50]	Low	Low	Low	Unclear	Low	Low	Low
Ko 2016 [48]	Low	Low	Low	Low	Low	Low	Low
Park 2016 [49]	Low	Low	Low	Low	Low	Low	Low
Yoon 2016 [15]	Low	Low	Low	Unclear	Low	Low	Low
Grani 2017 [51]	Low	Low	Low	Low	Low	Low	Low
Chng 2018 [16]	Low	Low	Low	Low	Low	Low	Low
Ha 2018 [11]	Low	Low	Low	Unclear	Low	Low	Low
Ahmadi 2019 [17]	Low	Low	Low	Low	Low	Low	Low
Gao 2019 [19]	Low	Low	Low	Unclear	Low	Low	Low
Li 2019 [20]	Low	Low	Low	Unclear	Low	Low	Low
Phuttharak 2019 [21]	Low	Low	Low	Low	Low	Low	Low
Shen 2019 [23]	Low	Low	Low	Unclear	Low	Low	Low
Shen 2019 [23]	Low	Low	Low	Low	Low	Low	Low
Wu 2019 [73]	Low	Low	Low	Unclear	Low	Low	Low
Wu 2019 [74]	Low	Low	Low	Low	Low	Low	Low
Zhao 2019 [52]	Low	Low	Low	Low	Low	Low	Low
Huang 2020 [24]	Low	Low	Low	Unclear	Low	Low	Low
Koc 2020 [25]	Low	Low	Low	Low	Low	Low	Low
Peng 2020 [26]	Low	Low	Low	Low	Low	Low	Low
Watkins 2020 [27]	Low	Low	Low	Unclear	Low	Low	Low
Xiao 2020 [28]	Low	Low	Low	Low	Low	Low	Low
Yang 2020 [29]	Low	Low	Low	Unclear	Low	Low	Low
Yoon 2020 [30]	Low	Low	Low	Low	Low	Low	Low
Zhang 2020 [75]	Low	Low	Low	Unclear	Low	Low	Low
Chen 2021 [47]	Low	Low	Low	Low	Low	Low	Low
da Silva 2021 [33]	Low	Low	Low	Low	Low	Low	Low
Ha 2021 [34]	Low	Low	Low	Unclear	Low	Low	Low
Han 2021 [35]	Low	Low	Low	Low	Low	Low	Low
Hekimsoy 2021 [36]	Low	Low	Low	Low	Low	Low	Low
Huh 2021 [37]	Low	Low	Low	Unclear	Low	Low	Low
Kang 2021 [38]	Low	Low	Low	Unclear	Low	Low	Low
Kim 2021 [53]	Low	Low	Low	Unclear	Low	Low	Low
Liu 2021 [54]	Low	Low	Low	Unclear	Low	Low	Low
Na 2021 [39]	Low	Low	Low	Low	Low	Low	Low
Qi 2021 [40]	Low	Low	Low	Unclear	Low	Low	Low
Scappaticcio 2021 [41]	Low	Low	Low	Unclear	Low	Low	Low
Seifert 2021 [42]	Low	Low	Low	Low	Low	Low	Low
Seminati 2021 [43]	Low	Low	Low	Unclear	Low	Low	Low
Sharma 2021 [55]	Low	Low	Low	Low	Low	Low	Low
Shi 2021 [44]	Low	Low	Low	Unclear	Low	Low	Low
Zhang 2021 [45]	Low	Low	Low	Low	Low	Low	Low
Zhu 2021 [46]	Low	Low	Low	Unclear	Low	Low	Low
Chen 2022 [32]	Low	Low	Low	Unclear	Low	Low	Low
Qi 2022 [6]	Low	Low	Low	Low	Low	Low	Low
Thedinger 2022 [76]	Low	Low	Low	Unclear	Low	Low	Low
Zhang 2022 [77]	Low	Low	Low	Low	Low	Low	Low
